# Ferrocene Bis(Sulfonate) Salt as Redoxmer for Fast and Steady Redox Flow Desalination

**DOI:** 10.3390/molecules29112506

**Published:** 2024-05-25

**Authors:** Rongxuan Xie, Briana R. Schrage, Junhua Jiang, Christopher J. Ziegler, Zhenmeng Peng

**Affiliations:** 1 Department of Chemical Engineering, University of South Carolina, Columbia, SC 29208, USA; rxie@email.sc.edu; 2 Department of Chemistry, The University of Akron, Akron, OH 44325, USA; brs69@uakron.edu; 3 Advanced and Energy Materials Department, Savannah River National Laboratory, Aiken, SC 29808, USA; junhua.jiang@srnl.doe.gov

**Keywords:** ferrocene, desalination, redoxmer

## Abstract

Desalination is considered a promising solution to alleviate water shortages, yet current methods are often restricted, due to challenges like high energy consumption, significant cost, or limited desalination capacity. In this study, we present a novel approach of redox flow desalination (RFD) utilizing the highly aqueous-soluble and reversible redox-active compound, potassium 1,1′-bis(sulfonate) ferrocene (1,1′-FcDS). This water-soluble organic compound yielded stable and rapid desalination, sustaining extended operation without notable decay and achieving an impressive desalination rate of up to 457.5 mmol·h^−1^·m^−2^ and energy consumption as low as 40.2 kJ·mol_NaCl_^−1^. Specifically, the RFD device effectively desalinated a 50 mM NaCl solution to potable standards within 6000 s using 1,1′-FcDS. It maintained an average energy consumption of 178.16 kJ·mol_NaCl_^−1^ and exhibited negligible deterioration in desalination rate, energy efficiency, and charge efficiency throughout a rigorous 12,000 s cycling test. Furthermore, the versatility of this method was demonstrated by effectively treating saline water with varying initial concentrations from 10 mM to 50 mM, showcasing its potential across a broad spectrum of applications.

## 1. Introduction

The escalating freshwater shortage presents an imminent crisis for humanity, exacerbated by rapid population growth [1,2]. Despite Earth’s abundant water resources, a staggering 97% exists in the form of seawater or saline water [3]. Current estimates indicate that one in three individuals lacks access to clean water sources, leading to a tragic toll of over 3.5 million deaths annually. Furthermore, freshwater scarcity will be a problem for over two-thirds of the global population by 2025, according to the projection [4].

In response to this crisis, desalination has emerged as a promising solution. Various desalination technologies, including evaporation, reverse osmosis, and electrodialysis, have been explored, yet their widespread application is hindered by significant drawbacks, such as prolonged operation time and high energy consumption [5,6,7,8]. Redox flow desalination (RFD), which harnesses solution-phase redox reactions in circulating aqueous electrolytes [9], has recently gained traction as a viable approach [10,11]. RFD offers compelling advantages, including high operational current, extended cycle life, flexibility in desalination capacity and rate scaling, and adaptability to diverse salinity levels, ranging from brackish to brine. However, the efficacy of RFD hinges on a critical component: redoxmers. These molecules, vital for enabling efficient and durable desalination, face challenges, such as instability [12], toxicity [13,14,15], and low aqueous solubility [16,17]. Consequently, there is a pressing need for the development of new redoxmers with high activity and stability. Addressing these imperfections is essential to unlock the full potential of RFD and to provide a sustainable solution to the impending global freshwater crisis.

Recently, there has been significant interest in ferrocene-based redox flow cells as a viable energy storage solution [18,19]. While initial investigations have explored the use of ferrocene materials in deionization [20,21,22,23], the predominant focus has been on capacitive deionization (CDI), which is constrained by its charge and discharge operational modes [24]. The potential of ferrocene materials in RFD, which is characterized by continuous operation and has theoretically unlimited capacity, remains largely unexplored. Notably, anionic and cationic ferrocenes have emerged as promising candidates, due to their high water solubility and reversible redox activity [19], making them ideal materials for RFD applications. In this study, we introduced potassium 1,1′-bis(sulfonate) ferrocene (1,1′-FcDS) as a redoxmer for the RFD cell design. Through comprehensive investigation, we optimized two crucial parameters to enhance desalination performance, thereby showcasing the remarkable potential of 1,1′-FcDS as a redoxmer in RFD systems.

## 2. Results and Discussion

**Figure 1:**
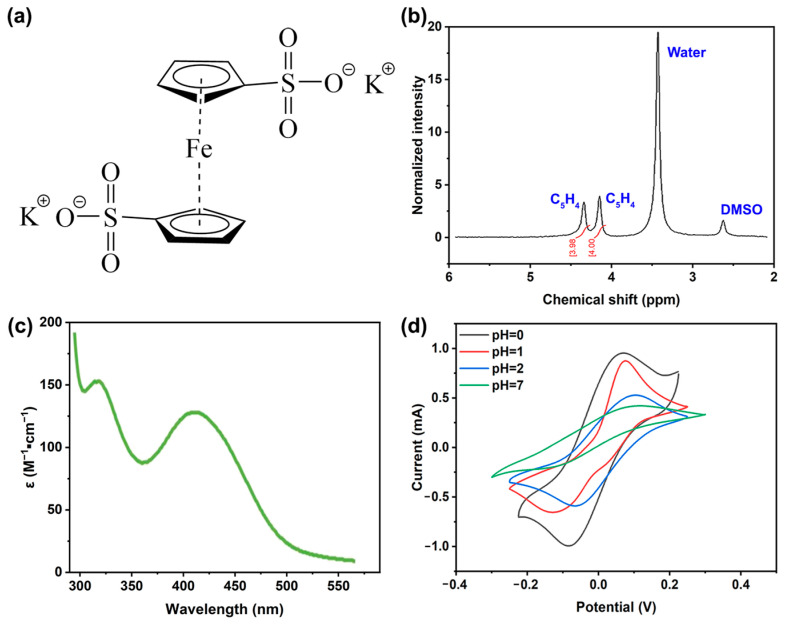
(**a**) Molecular structure of potassium 1,1′-bis(sulfonate) ferrocene (1,1′-FcDS), (**b**) 1H NMR (300 MHz) spectrum of 1,1′-FcDS in d6-DMSO, (**c**) UV-Vis spectrum of 1,1′-FcDS in water, and (**d**) CV curves of 50 mM 1,1′-FcDS at different pH in an aqueous electrolyte. Working electrode: 3 mm dia. glassy carbon, reference electrode: Ag|AgCl|KCl (1M), counter electrode: Pt wire.

Figure 1a depicts the molecular structure of 1,1′-FcDS, a ferrocene derivative featuring two peripheral sulfonate groups. This compound can be easily synthesized from readily available reagents [25]. In our study, we synthesized 1,1′-FcDS by sulfonating ferrocene and then neutralizing the resulting bis-sulfonic acid derivative to obtain the potassium salt. The inclusion of these anionic sulfonate functional groups plays a pivotal role in transforming the naturally hydrophobic ferrocene precursor into a significantly water-soluble compound. The synthesized sample was confirmed using NMR analysis (Figure 1b), revealing peaks at 4.12 ppm (4H on C_5_H_4_) and 4.33 ppm (4H on C_5_H_4_), providing conclusive evidence that 1,1′-FcDS was the sole product of the synthesis.

The aqueous solubility of 1,1′-FcDS was assessed through UV-Vis measurements, as illustrated in Figure 1c and Figure S1. The results demonstrated that 1,1′-FcDS exhibited remarkable solubility, reaching concentrations as high as 0.3 M. In the context of RFD application, the chemical stability of redoxmer is paramount for prolonged and reliable operation. Under acidic conditions, the aqueous solution of 1,1′-FcDS showed no discernible changes in appearance and maintained nearly 100% capacity (Figure S2). This stability can be attributed to the presence of hydronium ions, which prevented the air oxidation of Fe^2+^. To delve deeper into its electrochemical properties, cyclic voltammograms (CVs) were collected for 1,1′-FcDS, as presented in Figure 1d. When the compound was dissolved in an HCl solution with a pH of 0, it exhibited the highest current, indicating the fastest redox kinetics of 1,1′-FcDS. This further confirmed the role of H^+^ ions in preventing the oxidation of Fe^2+^ and promoting the electrochemical redox activity. In this work, we opted for an HCl solution with a pH of 1 for all subsequent experiments for safety and cell durability considerations.

**Figure 2:**
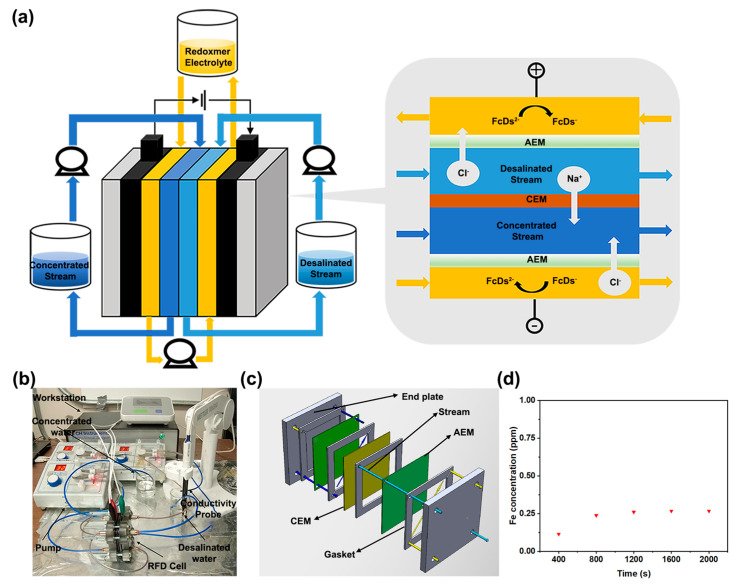
(**a**) Experimental set-up for the evaluation of desalination performance, (**b**) photo of the home-made RFD system, (**c**) schematic representations of the RFD cell, and (**d**) ICP-OEs results of Fe concentration in concentrated water reservoir versus time.

In the RFD system developed by our team and others (Figure 2a,b) [12,17,26,27,28], there are four main components, including the RFD cell, the redoxmer electrolyte reservoir, the concentrated water reservoir, and the desalinated water reservoir. Within the RFD cell (Figure 2b and Figure S3), a unique setup is employed. Instead of a single desalinated stream, parallel desalinated and concentrated streams, separated by a cation exchange membrane (CEM), are positioned between the anolyte and catholyte chambers, separated by an anion exchange membrane (AEM). The parameters and properties of employed ion exchange membranes are summarized in Table S1. These chambers are interconnected through flow tubing, facilitating the circulation of a redoxmer electrolyte between them. Within this setup, the electrolyte undergoes reduction in the catholyte chamber and oxidation in the anolyte chamber. This creates a charge disparity that prompts the migration of Na^+^ and Cl^–^ ions from the desalinated stream towards the concentrated streams, facilitated by cation exchange membranes (CEMs) and anion exchange membranes (AEMs). This mechanism enables the continuous removal of salt at low cell overpotentials, even with redoxmer concentrations at the millimolar scale. To assess whether there existed a crossover issue of 1,1′-FcDS from the electrolyte chambers through AEM to the water streams, samples were collected from the water streams after the RFD test and were analyzed for their Fe element using ICP-OES (Figure 2d). The results indicated that the concentration of 1,1′-FcDS was below 0.005 mM (<250 ppb), a value likely attributed to measurement error. This observation demonstrates that 1,1′-FcDS, due to its relatively large molecular size, cannot permeate through the AEM, highlighting a crucial aspect of its behavior within the RFD system.

**Figure 3:**
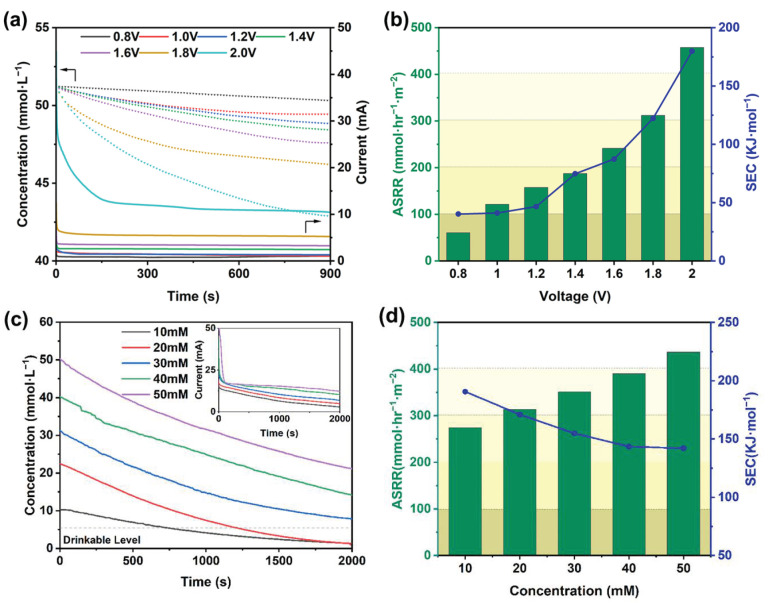
Effects of the applied voltage and initial saline concentration on RFD performance: (**a**) current (solid line) and conductivity (dotted line) profiles and (**b**) ASRR and SEC at different applied voltages, (**c**) current and conductivity profiles and (**d**) ASRR and SEC with saline water started with different NaCl concentrations. The flow rate was 25 mL·min^−1^.

An RFD cell utilizing an aqueous electrolyte containing 50 mM 1,1′-FcDS and 0.1 M HCl was assembled and operated to investigate crucial test parameters, notably applied cell voltage and the initial concentration of saline water. The influence of applied voltage, directly linked to energy input, was the first focus. Typically, the applied voltage is kept below 1.2 V to prevent side reactions like water electrolysis. However, considering the presence of internal resistance and the absence of an active catalyst for water electrolysis [29], the upper limit for applied voltage in this experiment was extended to 2.0 V. Using 50 mM NaCl solution as feedwater streams and a flow rate of 25 mL·min^−1^ for both the electrolyte flow and water streams flow, the RFD cell was tested under applied voltages ranging from 0.8 to 2.0 V. A higher applied voltage resulted in an increased RFD current and a faster decline in conductivity of the desalinated stream (Figure 3a and Figure S4). This is consistent with the RFD mechanism, in which the charge imbalance induced by the applied voltage serves as the primary driving force for ion migration [30]. Moreover, an increase in the average salt removal rate (ASRR) and specific energy consumption (SEC) was observed with rising applied voltage (Figure 3b).

The RFD cell showed a stable 0.8 mA current at 0.8 V, with 60.6 mmol·h^−1^·m^−2^ ASRR and 40.2 kJ·mol_NaCl_^−1^ SEC achieved over 900 s of experimentation. A significantly higher current of 13.7 mA was obtained when the applied voltage was increased to 2.0 V. Meanwhile, the ASRR and SEC changed to 457.5 mmol·h^−1^·m^−2^ and 179.8 kJ·mol_NaCl_^−1^, respectively. It is noteworthy that the ASRR and SEC properties already outperformed most literature results, especially when compared with previous studies of other flow electrode materials (Table S2), demonstrating the high redox activity of 1,1′-FcDS to drive desalination. Although a higher cell voltage largely improved the desalination rate, it can result in reduced Faradaic efficiency, due to undesired side reactions (Figure S5) and heightened energy consumption, due to increased ohmic losses. Striking a balance between two crucial performance metrics, ASRR and SEC, an applied voltage of 2.0 V was considered a worthy compromise and was thus employed in the subsequent study on the effect of initial concentration.

A pivotal characteristic of the RFD cell is its adaptability to treat saline water across a wide range of concentrations [31]. In this study, a NaCl solution spanning concentrations from 10 mM to 50 mM was examined to assess the efficacy of the 1,1′-FcDS-based RFD cell. Notably, the concentration of saline water plays a substantial role in determining the resistance within a flow cell, thereby directly impacting the performance of the RFD system. Higher concentrations of saline water are anticipated to result in reduced cell resistivity and an augmented desalination current. Figure 3c shows the changes in cell current and desalination stream concentration over time for desalinating the NaCl solution with different concentrations. An observable trend indicates a significant rise in current, i.e., a correspondingly higher ASRR, with an increasing NaCl concentration. Meanwhile, our analysis revealed that higher saline concentrations notably decreased SEC while simultaneously enhancing ASRR (Figure 3d). With a 10 mM NaCl solution, the RFD cell achieved an ASRR of 274 mmo·h^−1^·m^−2^ and an SEC of 190.7 kJ·mol_NaCl_^−1^. However, when the feed solution was 50 mM NaCl, the ASRR significantly increased to 436.4 mmol·h^−1^·m^−2^, accompanied by a decrease in SEC to 142.1 kJ·mol_NaCl_^−1^. This improvement can be attributed to an enhancement in the ion conductivity in the water stream as the solution concentration increases [32,33]. Consequently, more energy can be allocated to the migration of salt ions from the saline water stream to the concentrated stream.

**Figure 4:**
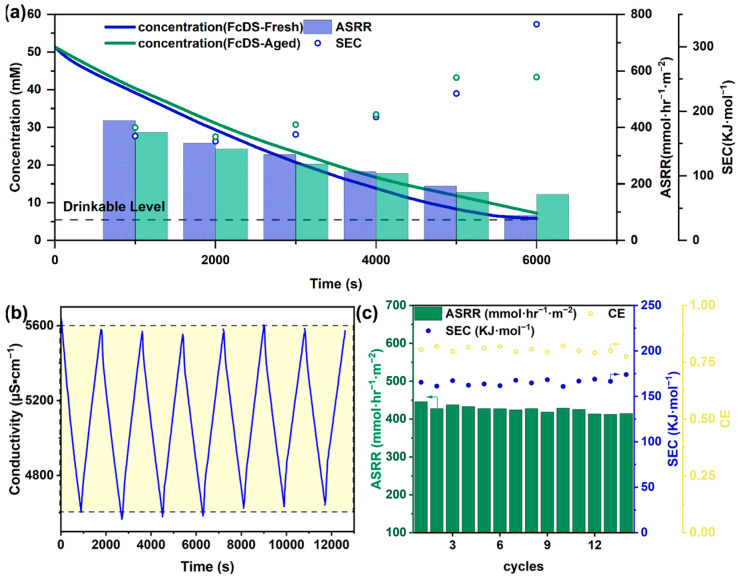
(**a**) Conductivity(lines), ASRR(colors), and SEC(dots) profiles as a function of time of RFD cells using fresh(blue) and aged(green) 1,1′-FcDS electrolytes, under an applied voltage of 2.0 V, and at a flow rate of 25 mL·min^−1^; (**b**) the desalinating–concentrating cycling profile of an RFD cell using a 1,1′-FcDS electrolyte, with a 25 mL·min^−1^ flow rate and ±2 V cell voltage flipped every 15 min; and (**c**) the cyclability performance evaluated by ASRR, SEC, and CE.

The stability of the 1,1′-FcDS electrolyte for RFD application was examined by desalinating a 50 mM NaCl saline solution all the way to a drinkable level (Figure 4a). The concentration profile over time demonstrated the continuous migration of Na^+^ and Cl^−^ ions out of the desalinated stream, rendering it portable within about 6000 s. Consistent with prior findings, the gradual decline in ASRR and increase in SEC, accompanied by the continuous concentration decrease, mirrored previous trends over time. To further assess the stability, we conducted repeat operations in the RFD using an aged electrolyte. Remarkably, the desalinated stream concentration, ASRR, and SEC profiles closely matched those obtained with the freshly prepared electrolyte, validating 1,1′-FcDS’s excellent stability under RFD conditions. The disparity in ASRR and SEC observed towards the operation’s end could be attributed to accumulated salt removal differences. The average SEC for our process stood at 178.16 kJ·mol_NaCl_^−1^, equivalent to approximately 2.45 kwh·m^−3^ of produced drinking water. This performance notably outperformed existing technologies like multiple effect distillation (MED, 15~57 kwh·m^−3^), multi-effect boiling (MEB, ~30 kwh·m^−3^), and multi-stage flash (MSF, 21~59 kwh·m^−3^), indicating the substantial economic advantage of our approach [34]. The RFD cell’s cyclability with the 1,1′-FcDS electrolyte was demonstrated by cyclic voltage flipping every 15 min (Figure 4b). This process reverses the electrolyte’s redox reactions at the electrodes, enabling the RFD to alternate between desalinating mode and concentrating mode. The cell exhibited stable performance and reproducible cyclability, with charge transfer amounts per cycle consistently falling within the range of 13 C to 14 C. Additionally, the saline water consistently reached similar desalination levels and returned to the initial concentration during concentrating cycles. The cyclability experiments affirmed the stability of both the 1,1′-FcDS electrolyte and the ion exchange membranes. These findings provide strong evidence that the use of 1,1′-FcDS does not lead to membrane-fouling issues under the operational conditions. Moreover, no noticeable changes in ASRR, SEC, and CE were observed during cycling operations or prolonged exposure to air (Figure 4c).

## 3. Materials and Methods

### 3.1. Materials

The redoxmer, potassium 1,1′-FcDS, was prepared from bis-ferrocene sulfonic acid, which was synthesized based on a previously reported procedure [35,36]. Sodium chloride, sodium hydroxide, and hydrochloric acid were purchased from commercial vendors and used without further purification. All ion exchange membranes used in this study were purchased from Fuel Cell Store (College Station, TX, USA).

### 3.2. Synthesis of 1,1′-FcDS

Bis-ferrocene sulfonic acid (5.0 g, 14.5 mmol) was dissolved in 200 mL of ethanol. Concentrated KOH solution was gradually added to the mixture while stirring for 30 min. The resulting yellow precipitate was filtered and allowed to air-dry, resulting in the formation of a yellow solid. The yield was 5.4 g (96%). The product was confirmed with ESI mass spectroscopy (calculated: 366.900, found: 366.9060), consistent with previous work [18].

### 3.3. 1,1′-FcDS Redox Flow Electrodes Preparation

Aqueous neutral and acidic solutions were prepared by dissolving 1,1′-FcDS (0.422 g, 0.001 mol) in 20 mL of solution and adding 0.234 g NaCl as the supporting electrolyte, resulting in yellow transparent solutions.

### 3.4. Salt Removal Tests

The RFD cell was equipped with two Fumasep FAS-PET-130 anion exchange membranes (AEMs) and one Fumasep FKS-PET-130 cation exchange membrane (CEM), providing a total active area of 9 cm². Desalination experiments were carried out in batches using a custom-built RFD system. This system circulated 20 mL of 1,1′-FcDS redoxmer solution and 15 mL of NaCl solution through the RFD cell using three peristaltic pumps. Voltage application and measurement were managed by a CHI 1140C electrochemical workstation. The conductivities of both desalinated and saline streams were monitored using a Mettler Toledo 230 conductivity meter equipped with an Inlab 741-ISM conductivity probe. The RFD’s performance was evaluated across varying initial NaCl concentrations (ranging from 10 mM to 50 mM) and applied voltages (ranging from 0.8 V to 2.0 V). All tests were conducted at room temperature.

The major desalination performance metrics were determined by Equations (1) and (2). The average salt removal rate (ASRR, in mmol·m^−2^·h^−1^) was calculated using Equation (1):

(1) $$ASRR=-\frac{\left(c-c_{0}\right)\times V_{NaCl}}{A\times t}$$

where c and c_0_ are the final and initial NaCl concentrations in the desalination stream, respectively, which were obtained from the conductivity-concentration calibration curve. V_NaCl_ is the total volume of the effluent stream (15 mL unless stated otherwise), A is the active area (9 cm^2^ in this work), and t is the desalination time.

The specific energy consumption (SEC, in kJ·mol_NaCl_^−1^) was calculated using Equation (2):

(2) $$SEC=\frac{V\times C}{\left(c-c_{0}\right)\times V_{NaCl}}$$

where V is the applied voltage, and C is the overall charge capacity during the desalination test, which can be obtained as the integral area of the corresponding current-time (I-t) plot.

The Charge efficiency was calculated using Equation (3):

(3) $$\mathrm{Charge}\;\mathrm{efficiency}=\frac{\left(c-c_{0}\right)\times V_{NaCl}}{I\times t}$$

where c and c_0_ are the final and initial NaCl concentrations in the desalination stream, respectively. They were obtained from the conductivity–concentration calibration curve. V_NaCl_ is the total volume of the saline water stream (15 mL unless stated otherwise), I is the desalination current, and t is the desalination time.

## 4. Conclusions

In summary, potassium 1,1′-bis(sulfonate) ferrocene (1,1′-FcDS) was synthesized and studied for its properties in water desalination applications. The 1,1′-FcDS exhibited a remarkable solubility in water, reaching as high as 0.3 M in concentration, and excellent electrochemical redox kinetics and stability, particularly in acidic electrolytes. When employed as a redoxmer in a redox flow desalination (RFD) cell system, efficient and durable desalination was achieved. The desalination performance was found to be a function of cell voltage and saline concentration. A higher cell voltage yielded a greater driving force, thereby enhancing the desalination rate, albeit at the expense of increased energy consumption due to heightened ohmic losses. The RFD cell demonstrated versability in treating saline water across concentrations ranging from 10 mM to 50 mM. Notably, higher saline concentrations significantly boosted the desalination rate from 274 mmo·h^−1^·m^−2^ to 436.4 mmol·h^−1^·m^−2^ while concurrently reducing energy consumption from 190.7 kJ·mol_NaCl_^−1^ to 142.1 kJ·mol_NaCl_^−1^, benefiting from improved ion conductivity. Specifically, an average salt removal rate (ASRR) of 457.5 mmol·h^−1^·m^−2^ with 179.8 kJ·mol_NaCl_^−1^ specific energy consumption (SEC) was achieved using a cell voltage of 2 V to treat 50 mM NaCl solution. Repetitive operation and cyclability experiments showed a stable and reproducible desalination performance, confirming the excellent stability of 1,1′-FcDS redoxmer under the RFD conditions. The research findings in this work validate the interesting properties of 1,1′-FcDS redoxmer and its potential for use as an electrolyte for efficient water desalination applications.

## Data Availability

Data are contained within the article and Supplementary Materials.

## Supplementary Materials

The following supporting information can be downloaded at: https://www.mdpi.com/article/10.3390/molecules29112506/s1, Figure S1: Solubility measurements using UV-vis spectroscopy; Figure S2: Photos and CVs of potassium 1,1′ FcDS; Figure S3: Photo of the RFD cell structure; Figure S4: The conductivity–concentration calibration curve for NaCl solutions; Figure S5: Effects of applied voltage on Faraday efficiency. Table S1: The main characteristics of the ion exchange membranes used in this research; Table S2: Comparison of desalination performances of various materials from literature. References [37,38,39,40,41,42,43,44,45,46] are cite in the Supplementary Materials.

## References

[1] Karagiannis I.C., Soldatos P.G. (2008). Water desalination cost literature: Review and assessment. Desalination.

[2] Al-Rajabi M.M., Abumadi F.A., Laoui T., Atieh M.A., Khalil K.A. (2024). Capacitive deionization for water desalination: Cost analysis, recent advances, and process optimization. J. Water Process Eng..

[3] Darre N.C., Toor G.S. (2018). Desalination of Water: A Review. Curr. Pollut. Rep..

[4] Mekonnen M.M., Hoekstra A.Y. (2016). Four billion people facing severe water scarcity. Sci. Adv..

[5] Duan F., Du X., Li Y., Cao H., Zhang Y. (2015). Desalination stability of capacitive deionization using ordered mesoporous carbon: Effect of oxygen-containing surface groups and pore properties. Desalination.

[6] Boota M., Hatzell K.B., Alhabeb M., Kumbur E.C., Gogotsi Y. (2015). Graphene-containing flowable electrodes for capacitive energy storage. Carbon.

[7] Rommerskirchen A., Kalde A., Linnartz C.J., Bongers L., Linz G., Wessling M. (2019). Unraveling charge transport in carbon flow-electrodes: Performance prediction for desalination applications. Carbon.

[8] Bouhadana Y., Ben-Tzion M., Soffer A., Aurbach D. (2011). A control system for operating and investigating reactors: The demonstration of parasitic reactions in the water desalination by capacitive de-ionization. Desalination.

[9] Weber A.Z., Mench M.M., Meyers J.P., Ross P.N., Gostick J.T., Liu Q. (2011). Redox flow batteries: A review. J. Appl. Electrochem..

[10] Desai D., Beh E.S., Sahu S., Vedharathinam V., van Overmeere Q., de Lannoy C.F., Jose A.P., Völkel A.R., Rivest J.B. (2018). Electrochemical Desalination of Seawater and Hypersaline Brines with Coupled Electricity Storage. ACS Energy Lett..

[11] Hou X., Liang Q., Hu X., Zhou Y., Ru Q., Chen F., Hu S. (2018). Coupling desalination and energy storage with redox flow electrodes. Nanoscale.

[12] Xie R., Yue D., Peng Z., Wei X. (2023). Achieving Energy-Saving, Continuous Redox Flow Desalination with Iron Chelate Redoxmers. Energy Mater. Adv..

[13] Scialdone O., Guarisco C., Grispo S., Angelo A.D., Galia A. (2012). Investigation of electrode material—Redox couple systems for reverse electrodialysis processes. Part I: Iron redox couples. J. Electroanal. Chem..

[14] Liang M., Zhang J., Ramalingam K., Wei Q., San Hui K., Htike Aung S., Nam Hui K., Chen F. (2022). Stable and efficient self-sustained photoelectrochemical desalination based on CdS QDs/BiVO_4_ heterostructure. Chem. Eng. J..

[15] Alkhaldi A., Alsultan A., Alsaikhan K., Xie R., Li J., Peng Z. (2023). Removal and Recovery of Ammonium and Phosphate from Wastewater Using a Redox Flow Deionization Cell (RFDC). ACS EST Water.

[16] Lu D., Xu C., Wang Y., Cai W. (2022). Continuous desalination via redox flow desalination using sodium 4-sulfonatooxy-2,2,6,6-tetramethyl-piperidine-1-oxyl (NaSO4-TEMPO). Chem. Eng. J..

[17] Pan Y., Yao L., Wu D., Bentalib A., Li J., Peng Z. (2020). Sulfonated nickel phthalocyanine redox flow cell for high-performance electrochemical water desalination. Desalination.

[18] Zhao Z., Zhang B., Schrage B.R., Ziegler C.J., Boika A. (2020). Investigations into Aqueous Redox Flow Batteries Based on Ferrocene Bisulfonate. ACS Appl. Energy Mater..

[19] Li Y., Xu Z., Liu Y., Jin S., Fell E.M., Wang B., Gordon R.G., Aziz M.J., Yang Z., Xu T. (2021). Functioning Water-Insoluble Ferrocenes for Aqueous Organic Flow Battery via Host–Guest Inclusion. ChemSusChem.

[20] Gao F., Li X., Shi W., Wang Z. (2022). Highly Selective Recovery of Phosphorus from Wastewater via Capacitive Deionization Enabled by Ferrocene-polyaniline-Functionalized Carbon Nanotube Electrodes. ACS Appl. Mater. Interfaces.

[21] Song Z., Garg S., Ma J., Waite T.D. (2020). Selective Arsenic Removal from Groundwaters Using Redox-Active Polyvinylferrocene-Functionalized Electrodes: Role of Oxygen. Environ. Sci. Technol..

[22] Nwokonkwo O., Pelletier V., Broud M., Muhich C. (2023). Functionalized Ferrocene Enables Selective Electrosorption of Arsenic Oxyanions over Phosphate—A DFT Examination of the Effects of Substitutional Moieties, pH, and Oxidation State. J. Phys. Chem. A.

[23] Gao F., Shi W., Wang Z. (2022). Selective Phosphorus Removal from Wastewater Using Graphene Aerogel Loaded with Ferrocene-Polyaniline: Synergetic Adsorption and Electrochemically Mediated Oxidation. ACS EST Water.

[24] Sayed E.T., Obaid M., Olabi A.G., Abdelkareem M.A., Al Radi M., Al-Dawoud A., Al-Asheh S., Ghaffour N. (2023). Recent progress on the application of capacitive deionization for wastewater treatment. J. Water Process Eng..

[25] Schrage B.R., Zhao Z., Boika A., Ziegler C.J. (2019). Evaluating ferrocene ions and all-ferrocene salts for electrochemical applications. J. Organomet. Chem..

[26] Beh E.S., Benedict M.A., Desai D., Rivest J.B. (2019). A Redox-Shuttled Electrochemical Method for Energy-Efficient Separation of Salt from Water. ACS Sustain. Chem. Eng..

[27] Chen F., Wang J., Feng C., Ma J., David Waite T. (2020). Low energy consumption and mechanism study of redox flow desalination. Chem. Eng. J..

[28] Liang M., Feng K., Karthick R., Zhang L., Shi Y., Hui K.S., Hui K.N., Jiang F., Chen F. (2020). Photocathode-assisted redox flow desalination. Green Chem..

[29] Luo L., He Q., Ma Z., Yi D., Chen Y., Ma J. (2021). In situ potential measurement in a flow-electrode CDI for energy consumption estimation and system optimization. Water Res..

[30] Huang C., Sun J., Wang C., Zhang Q., Wang M., Zhang P., Xue Z., Jing Y., Jia Y., Shao F. (2022). Lithium Isotope Electromigration Separation in an Ionic Liquid–Crown Ether System: Understanding the Role of Driving Forces. Ind. Eng. Chem. Res..

[31] Wang Z., Wu Q., Li J., Qiu S., Cao D., Xu Y., Liu Z., Yu X., Sun Y. (2017). Two benzoyl coumarin amide fluorescence chemosensors for cyanide anions. Spectrochim. Acta Part A Mol. Biomol. Spectrosc..

[32] Chen Z., Song C., Sun X., Guo H., Zhu G. (2011). Kinetic and isotherm studies on the electrosorption of NaCl from aqueous solutions by activated carbon electrodes. Desalination.

[33] García-Quismondo E., Santos C., Soria J., Palma J., Anderson M.A. (2016). New Operational Modes to Increase Energy Efficiency in Capacitive Deionization Systems. Environ. Sci. Technol..

[34] Stillwell A.S., Webber M.E. (2016). Predicting the Specific Energy Consumption of Reverse Osmosis Desalination. Water.

[35] Knox G.R., Pauson P.L. (1958). 134. Ferrocene derivatives. Part VII. Some sulphur derivatives. J. Chem. Soc..

[36] Chanawanno K., Holstrom C., Crandall L.A., Dodge H., Nemykin V.N., Herrick R.S., Ziegler C.J. (2016). The synthesis and structures of 1,12032-bis(sulfonyl)ferrocene derivatives. Dalton Trans..

[37] FumaTech (2021). Technical Datasheet—Fumasep® FKS-PET-130. https://www.bwt.com/en/-/media/bwt/fumatech/datasheets/new/fumasep/water-treatment-processes/fumasep-fkspet130-dry-formv22.pdf?rev=1916492ac05d40debe2357b9c25e7c48.

[38] FumaTech (2021). Technical Datasheet—Fumasep® FAS-PET-130. https://www.bwt.com/en/-/media/bwt/fumatech/datasheets/new/fumasep/water-treatment-processes/fumasep-faspet130-dry-formv22.pdf?rev=d8a5924b6427472289b96e8babd3f489.

[39] You D., Feng Z., Wu J., Xiao Z., Li X., Yu Y. (2024). Highly permselective and conductive composite anion exchange membranes QPT@PE for electrodialysis desalination. Desalination.

[40] Fan H., Xu Y., Zhao F., Chen Q.-B., Wang D., Wang J. (2023). A novel porous asymmetric cation exchange membrane with thin selective layer for efficient electrodialysis desalination. Chem. Eng. J..

[41] Ma J., Shen G., Zhang R., Niu J., Zhang J., Wang X., Liu J., Li X., Liu C. (2022). Small particle size activated carbon enhanced flow electrode capacitive deionization desalination performances by reducing the interfacial concentration difference. Electrochim. Acta.

[42] Li Y., Yong T., Qi J., Wu J., Lin R., Chen Z., Li J. (2023). Enhancing the electronic and ionic transport of flow-electrode capacitive deionization by hollow mesoporous carbon nanospheres. Desalination.

[43] Ma J., He D., Tang W., Kovalsky P., He C., Zhang C., Waite T.D. (2016). Development of Redox-Active Flow Electrodes for High-Performance Capacitive Deionization. Environ. Sci. Technol..

[44] Xu L., Peng S., Mao Y., Zong Y., Zhang X., Wu D. (2022). Enhancing Brackish Water Desalination using Magnetic Flow-electrode Capacitive Deionization. Water Res..

[45] Zhang Q., Aung S.H., Oo T.Z., Chen F. (2020). Continuous electrochemical deionization by utilizing the catalytic redox effect of environmentally friendly riboflavin-5′-phosphate sodium. Mater. Today Commun..

[46] Wang J., Zhang Q., Chen F., Hou X., Tang Z., Shi Y., Liang P., Yu D.Y.W., He Q., Li L.-J. (2019). Continuous desalination with a metal-free redox-mediator. J. Mater. Chem. A.

