# Supplementary material for: Ferrocene Bis(Sulfonate) Salt as Redoxmer for Fast and Steady Redox Flow Desalination
Source: Molecules. 2024 May 25;29(11):2506. doi: 10.3390/molecules29112506 (PMC11173638; doi:10.3390/molecules29112506)
Supplement: Supplementary file 1 [file molecules-29-02506-s001.zip › molecules-3002904-supplementary.pdf]

Supplementary Information for

Ferrocene Bis(sulfonate) Salt as Redoxmer for Fast and Steady Redox Flow Desalination

Rongxuan Xie [1], Briana R. Schrage [2], Junhua Jiang [3], Christopher J. Ziegler [2,*] and Zhenmeng Peng [1,*]

[1] Department of Chemical Engineering, University of South Carolina, Columbia, SC 29208, USA

[2] Department of Chemistry, The University of Akron, Akron, OH 44325, USA

[3] Advanced and Energy Materials Department, Savannah River National Laboratory, Aiken, SC 29808, USA

AUTHOR INFORMATION

Corresponding Authors

* Christopher J. Ziegler: ziegler@uakron.edu

* Zhenmeng Peng: zmpeng@sc.edu

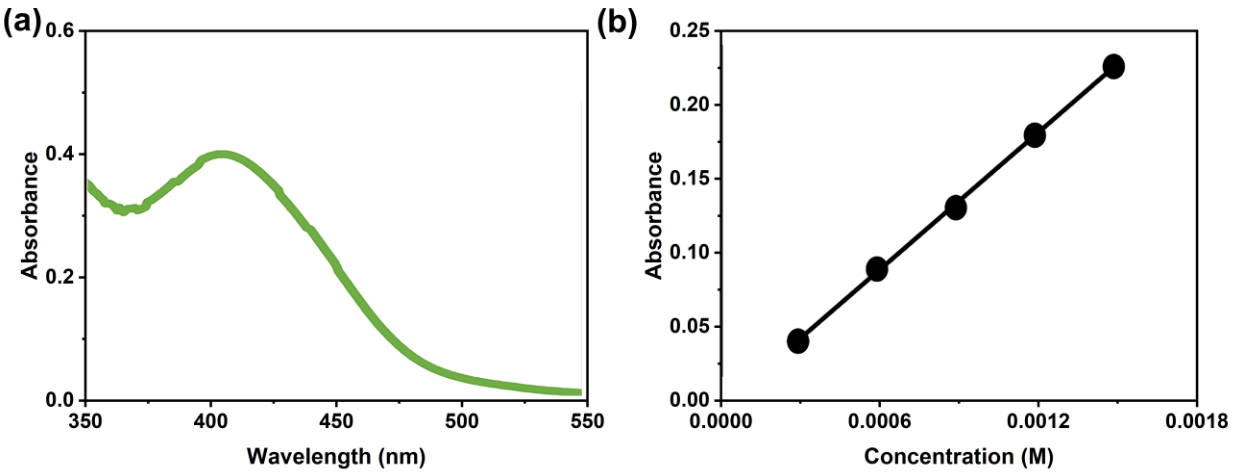

Figure. S1. Solubility measurements using UV–vis spectroscopy (a) UV–vis spectra of diluted supernatant of 1,1′-FcDS supersaturated solutions (dilution 200 times), and (b) calibration curves for therelationship between absorbance and concentration of 1,1′-FcDS.

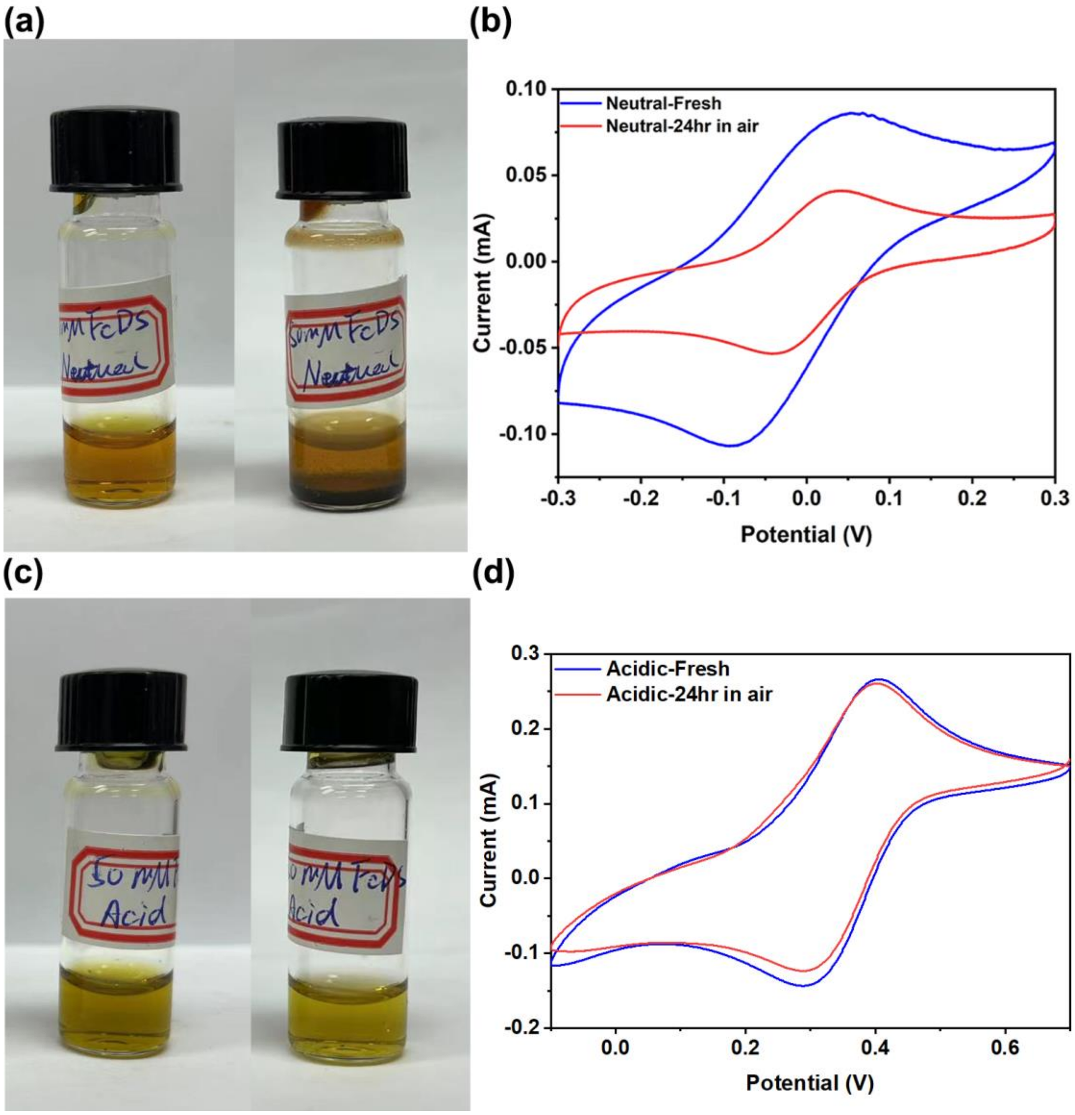

Figure. S2. (a) Photos of fresh (left) and aged (right) potassium 1,1′ FcDS in a neutral aqueous solution; (b) CV of 20mM potassium 1,1′-FcDS in a neutral aqueous solution. Working electrode: 3 mm dia. glassy carbon, reference electrode: Ag|AgCl|KCl (1M), counter electrode: platinum wire, (c) photos of fresh (left) and aged (right) potassium 1,1′-FcDS in a 0.01M HCl aqueous solution; (d) CV of 20 mM potassium 1,1′-FcDS in a 0.01M HCl aqueous solution. Working electrode: 3 mm dia. glassy carbon, reference electrode: Ag|AgCl|KCl (1M), counter electrode: platinum wire.

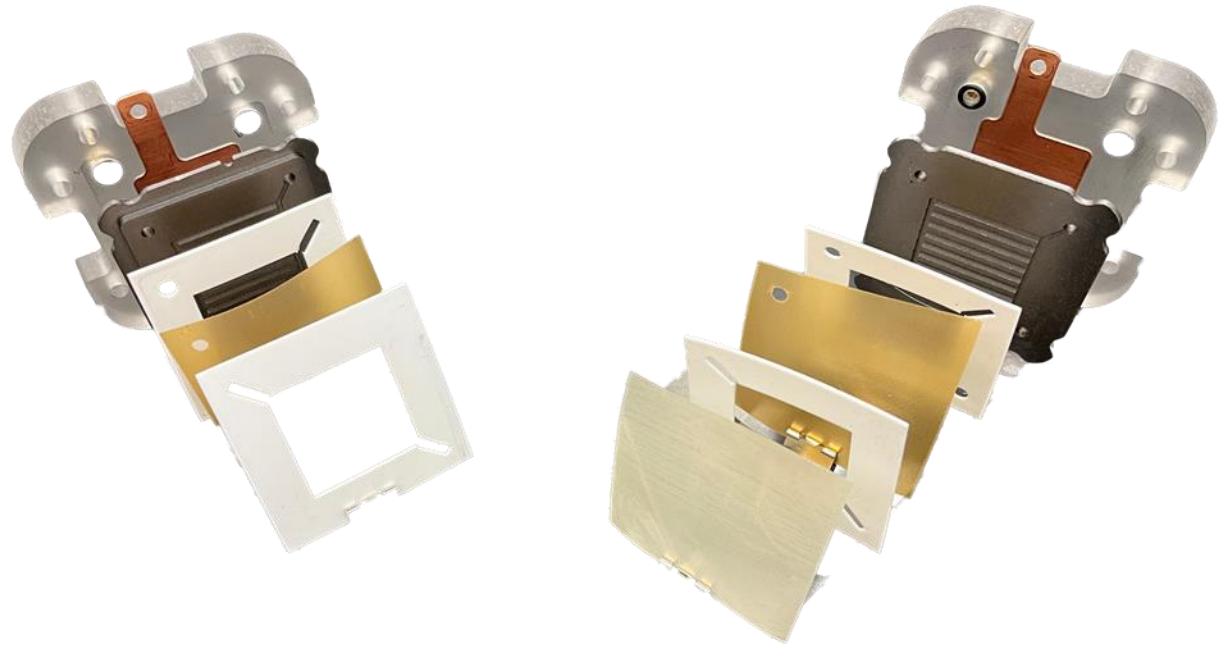

Figure. S3. Photo of the RFD cell structure.

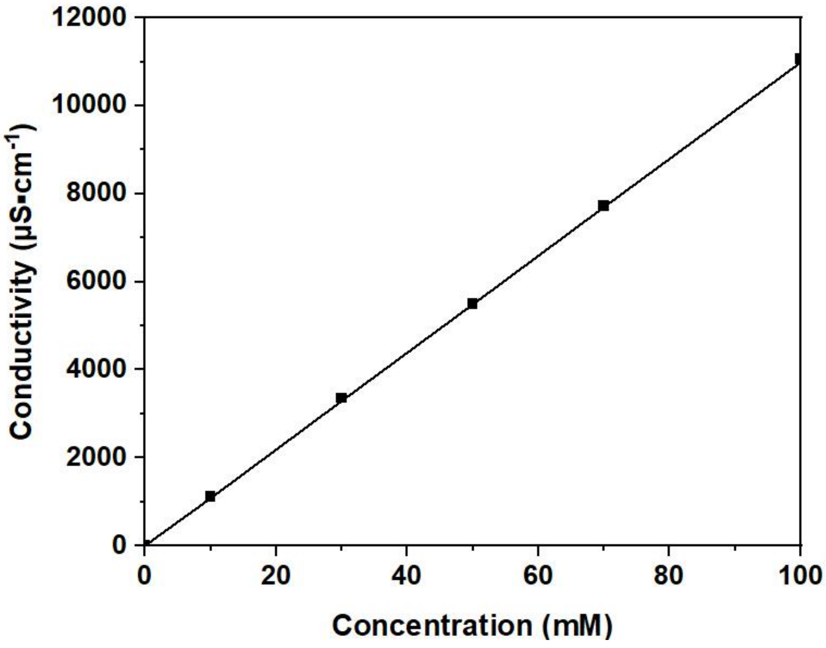

Figure. S4. The conductivity-concentration calibration curve for NaCl solutions.

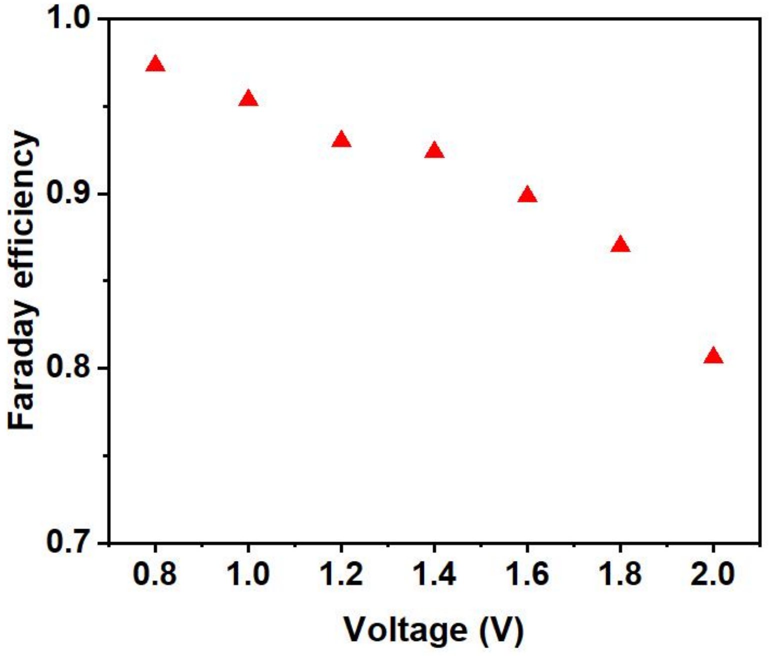

Figure. S5. Effects of applied voltage on Faraday efficiency.

Table S1. The main characteristics of the ion exchange membranes used in this research.

Materials	FKS-PET-130[1]	FAS-PET-130[2]
Membrane type	0.1	Anion exchange membrane
Counter ion	H^+ form	bromide (Br^-)
Thickness (dry, μm)	120 – 150	120 – 150
Weight per unit area (dry, $g{\cdot}m^{-2}$)	120 – 160	110 – 160
Area resistance($\Omega{\cdot}cm^2$)	< 4	< 4
Dimensional swelling in H_2O (%)	< 2	< 2
pH stability range	0 – 8	0 – 8
Operational temperature range (°C)	15 - 40	15 - 40

Table S2. Comparison of desalination performance of various materials from literature.

Materials	Type	Initial concentration (g/L)	ASRR (mmol▪ hr^{-1}▪m^{-2})	SEC (kJ▪mol^{-1})
QPT@PE [3]	ED	29.25	5032	465.9
SPES-PES [4]	ED	5.85	460	1148.3
BAC [5]	RFD	0.4	192	62.5
HMCNs [6]	RFD	1	317.9	117.6
AC DARCO [7]	RFD	2	252	69.4
MAC [8]	RFD	5	270	136.8
$[FMN]^{1/2-}$ [9]	RFD	5	135.1	180
$[TEMPO]^{0/+}$ [10]	RFD	5	145.3	129.12
1,1′-FcDS (this work)	RFD	3	457.5	100.2

References

1. FumaTech. *Technical Datasheet - fumasep® FKS-PET-130*. 2021. https://www.bwt.com/en/-/media/bwt/fumatech/datasheets/new/fumasep/water-treatment-processes/fumasep-fkspet130-dry-formv22.pdf?rev=1916492ac05d40debe2357b9c25e7c48.

2. FumaTech. *Technical Datasheet - fumasep® FAS-PET-130*. 2021. https://www.bwt.com/en/-/media/bwt/fumatech/datasheets/new/fumasep/water-treatment-processes/fumasep-faspet130-dry-formv22.pdf?rev=d8a5924b6427472289b96e8babd3f489.

3. You, D.; Feng, Z.; Wu, J.; Xiao, Z.; Li, X.; Yu, Y. Highly permselective and conductive composite anion exchange membranes QPT@PE for electrodialysis desalination. *Desalination* **2024**, *574*, 117240.

4. Fan, H.; Xu, Y.; Zhao, F.; Chen, Q.-B.; Wang, D.; Wang, J. A novel porous asymmetric cation exchange membrane with thin selective layer for efficient electrodialysis desalination. *Chemical Engineering Journal* **2023**, *472*, 144856.

5. Ma, J.; Shen, G.; Zhang, R.; Niu, J.; Zhang, J.; Wang, X.; Liu, J.; Li, X.; Liu, C. Small particle size activated carbon enhanced flow electrode capacitive deionization desalination performances by reducing the interfacial concentration difference. *Electrochimica Acta* **2022**, *431*, 140971. DOI:

6. Li, Y.; Yong, T.; Qi, J.; Wu, J.; Lin, R.; Chen, Z.; Li, J. Enhancing the electronic and ionic transport of flow-electrode capacitive deionization by hollow mesoporous carbon nanospheres. *Desalination* **2023**, *550*, 116381.

7. Ma, J.; He, D.; Tang, W.; Kovalsky, P.; He, C.; Zhang, C.; Waite, T. D. Development of Redox-Active Flow Electrodes for High-Performance Capacitive Deionization. *Environmental Science & Technology* **2016**, *50* (24), 13495-13501.

8. Xu, L.; Peng, S.; Mao, Y.; Zong, Y.; Zhang, X.; Wu, D. Enhancing Brackish Water Desalination using Magnetic Flow-electrode Capacitive Deionization. *Water Research* **2022**, *216*, 118290.

9. Zhang, Q.; Aung, S. H.; Oo, T. Z.; Chen, F. Continuous electrochemical deionization by utilizing the catalytic redox effect of environmentally friendly riboflavin-5'-phosphate sodium. *Materials Today Communications* **2020**, *23*, 100921.

10. Wang, J.; Zhang, Q.; Chen, F.; Hou, X.; Tang, Z.; Shi, Y.; Liang, P.; Yu, D. Y. W.; He, Q.; Li, L.-J. Continuous desalination with a metal-free redox-mediator. *Journal of Materials Chemistry A* **2019**, *7* (23), 13941-13947, 10.1039/C9TA02594D.
